# Insights From Liquid Chromatography-Mass Spectrometry-Measured Androgens in Indian Women With Polycystic Ovary Syndrome

**DOI:** 10.7759/cureus.53258

**Published:** 2024-01-30

**Authors:** Karthik Subramaniam, Nimmi Kansal

**Affiliations:** 1 Endocrinology, Silverline Hospital, Kochi, IND; 2 Pathology, Dr Lal Path Labs, Delhi, IND

**Keywords:** androstenedione, pcos phenotypes, androgen profile, dehydroepiandrosterone, testosterone

## Abstract

Introduction: Hyperandrogenemia is the defining feature of polycystic ovary syndrome (PCOS). Increasingly androgens are being advocated to be measured through liquid chromatography-mass spectrometry (LC-MS/MS). The role of LC-MS/MS over immunoassay in diagnosis of PCOS has been debated over a long time. We analyzed the role of androgens as measured by LC-MS/MS in diagnosing women with PCOS.

Materials and methods: We performed a prospective case-control study involving 59 women with PCOS compared with 30 age- and BMI-matched controls.

Results: In PCOS phenotypes A-C (hyperandrogenic by definition), 19/47 (40%) had normal testosterone (T) levels but 14/19 (75%) had either elevated androstenedione (A4) or dehydroepiandrosterone. A4 had the highest area under curve (0.89) for diagnosing PCOS followed by T (0.81). Even in the PCOS-D phenotype (sonologic polycystic ovaries + oligomenorrhoea), A4 was significantly higher as compared to controls though still in normal range.

Conclusion: A4 had a role in diagnosing hyperandrogenism in women with PCOS. Further studies clarifying the role of androgen profiles in diagnosing PCOS and its cost-effectiveness may be required in the future.

## Introduction

Polycystic ovary syndrome (PCOS) is the most common endocrine disorder in women of reproductive age. Hyperandrogenism is the hallmark feature of PCOS along with insulin resistance. Hyperandrogenism can be either clinically manifested as cystic acne, male pattern hair loss, female pattern balding, hirsutism and in severe forms as virilization or it can be detected biochemically. Most commonly studied circulating androgen in women is testosterone (T), but several other clinically important and less recognized hormones like androstenedione (A4), dihydrotestosterone (DHT), 11 keto steroids, dehydroepiandrosterone (DHEA), dehydroepiandrosterone sulphate (DHEAS), 17 hydroxy progesterone (17OHP) have also been studied. Several of these androgens ultimately get converted directly or through a back door pathway to T or DHT for its final effect in tissues.

Immunoassays work on the principle of measuring the analyte (usually antigen) using antigen-antibody complex that is formed in due course of the detection process. Steroids inherently form poor epitopes and hence have cross-reactivity and poor reproducibility while measuring them through immunoassays [1]. For example, thyroid stimulating hormone (TSH) has a cross-reactivity of 0.038% with luteinizing hormone (LH) though both share a subunit but testosterone has a cross-reactivity of ~2.5% with A4 and 0.1% with estradiol (Roche Diagnostics GmbH, Mannheim, Germany). Another aspect of hyperandrogenism that has implications in laboratory measurement is that of sex hormone binding globulin (SHBG). SHBG is low (and hence total T is also low) in such states and since assays of free T are much more unreliable, it makes a case for more accurate measurement of total T. Liquid chromatography with mass spectrometry (LC-MS/MS) has been shown not only to be highly sensitive and accurate to low quantities of androgens but also useful in steroid profiling. For example, the functional sensitivity of one of the T immunoassays is 21 ng/dL (Roche Diagnostics GmbH), while that in LC-MS/MS is 0.5 ng/dL (assay used in this study).

Recent PCOS guideline advocates the use of measuring A4 and DHEAS if T/free T values are normal in the face of high suspicion. This becomes much more important in women with minimal or no signs of hyperandrogenism [2]. The suggestion to use LC-MS/MS for this purpose is idealistic but practical difficulties in the form of cost-effectiveness and accessibility remain a problem. Previous research has shown that T and A4 done in isolation or as a panel of steroids have only a slight improvement in detecting hyperandrogenism and in the diagnosis of PCOS [3-5]. With this knowledge, we wanted to see the performance of the androgen panel measured by LC-MS/MS in diagnosing PCOS in Indian women.

## Materials and methods

This was a prospective case-control study over a duration of three years (2019-2022). It was performed in an outpatient setting of an endocrine clinic in Kerala, South India. Women aged above 20 years diagnosed with PCOS were recruited after written consent. PCOS was diagnosed based on Rotterdam criteria and phenotypes were classified as described [6]. Pregnant women, women on oral contraceptive pills, and women with endocrine disorders (hyperthyroidism, Cushing syndrome, hyperprolactinemia, congenital adrenal hyperplasia (CAH)) were excluded. Controls were chosen after consent from women of similar groups who had regular periods, no hirsutism, and normal ultrasound ovary features from the health check-up department.

Blood samples were collected in the follicular phase of the cycle. The serum was separated and transported to a national reference lab for LC-MS/MS analysis. The reagents and standards were procured from Chromsystem MassChrom® steroids (Chromsystems Instruments and Chemicals GmbH, Munich, Germany). LC-MS/MS was performed using SCIEX QTRAP 4500 system coupled with ExionLC (AB Sciex Pte Ltd, Framingham, MA, USA). Limits of quantification for androgens were T - 0.5 ng/dL, A4 - 23 ng/L, DHEA - 0.23 ng/mL, DHEAS - 24.4 ng/mL, 17OHP - 0.04 ng/ml. Upper limits of normal were T > 45 ng/dL, A4 > 2500 ng/L, DHEA > 9.8 ng/mL, DHEAS > 5270 ng/mL and 17OHP > 2 ng/mL [7].

Statistics

Based on a previous study, with 95% CI and 5% alpha error and case/control ratio of 2/1 along with a mean difference of 0.3 ng/ml and SD of 0.6 ng/ml for A4, case to controls were calculated as 96 to 48 respectively [3]. However, interim analysis was done when cases to controls numbers reached 60:30 respectively. Since the study was not funded, in order to reduce the research cost, recruitment was stopped at that stage as the power of study was >90%. The data were checked for normality using the Shapiro-Wilk test. Because the majority of results were not normal, Mann-Whitney U was used for comparing groups. Spearman’s correlation was used for correlating between androgens. Receiver operating characteristic (ROC) curve analysis was done to determine the best cutoff for diagnosing PCOS status. Data were analyzed using Statistical Package for Social Sciences (SPSS) version 26.0 (IBM Corp., Armonk, NY, USA). Level of significance to reject the null hypothesis was 5%.

## Results

The proportion of women with different phenotypes of PCOS were 26 (44%) with A, 13 (22%) with B, eight (14%) with C and 12 (20%) with D. When compared to age- and BMI-matched controls, PCOS women had high levels of all androgens except DHEAS (Table 1). Four women had 17OHP levels > 2 ng/ml warranting evaluation for CAH. Stimulated 17OHP were < 10 ng/ml in both women. 

**Table 1:** Pattern of hyperandrogenemia in polycystic ovary syndrome (PCOS) women and controls Data are presented as median (interquartile range). * p<0.01 between PCOS versus controls # p<0.01 between PCOS A-C versus PCOS D & p<0.01 between PCOS A-C versus controls $ p<0.05 between PCOS D versus controls 17OHP – 17 hydroxyprogesterone, A4 – androstenedione, BMI – body mass index, DHEA – dehydroepiandrosterone, DHEAS – dehydroepiandrosterone sulfate, T – testosterone

Parameter	Total PCOS (N=59)	PCOS A-C (N=47)	PCOS D (N=12)	Controls (N=30)
Age in years	23 (7)	24 (7)	21 (3.7)	25 (11.5)
BMI in kg/m^2^	25 (9)	26 (9)	24.5 (7)	24.5 (6)
A4 in ng/L	2430 (1460)^*^	2540 (1430)^#&^	1510 (795)^$^	1195 (640)
T in ng/dL	45 (45)^*^	50 (42)^#&^	29 (11)	25 (12)
DHEA in ng/mL	5.6 (8.5)^*^	8.9 (7.9)^#&^	2.9 (2.7)	3.3 (2.8)
DHEAS in ng/mL	1470 (1644)	1480 (1809)	1510 (1594)	1680 (1271)
17OHP in ng/mL	0.7 (0.59)^*^	0.77 (0.7)^#&^	0.64 (0.43)	0.37 (0.35)

**Figure 1:**
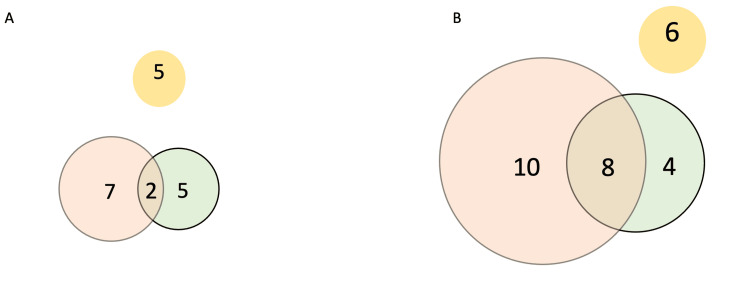
Overlapping pattern of androgens in polycystic ovary syndrome (PCOS) phenotypes Venn diagram denoting two groups – (A) PCOS A-C women with testosterone ≤ 45 ng/dL and (B) PCOS A-C women with testosterone > 45 ng/dL. Yellow color denotes those with normal androstenedione (A4) and dehydroepiandrosterone (DHEA) levels. Light red denotes those with increased A4 levels. Light green denotes those with increased DHEA levels.

Using a cut-off of ≤ 45 ng/dL as normal T, women with hyperandrogenism were classified into two groups. Nineteen of 47 (~40%) had normal T values. Only five out of those 19 with normal T had no elevation in other androgens and the remaining 14 had various combinations of androgen increase (Figure 1). On the contrary, only 6/28 had normal levels of A4 and DHEA in spite of high T. 

Since hyperandrogenism is common in PCOS A, B and C phenotypes, comparisons clubbing them as single group were also done. Except for DHEAS, all other androgens were significantly different in the PCOS A-C group versus controls who were age- and BMI-matched. In the PCOS A-C subgroup, A4 was significantly correlated with 17OHP levels (r = 0.4, p = 0.008) and DHEA was correlated with DHEAS (r = 0.7, p=0.00). In the PCOS D group, which did not have hyperandrogenemia, A4 was significantly different even in the normal range when compared to controls. 

**Figure 2:**
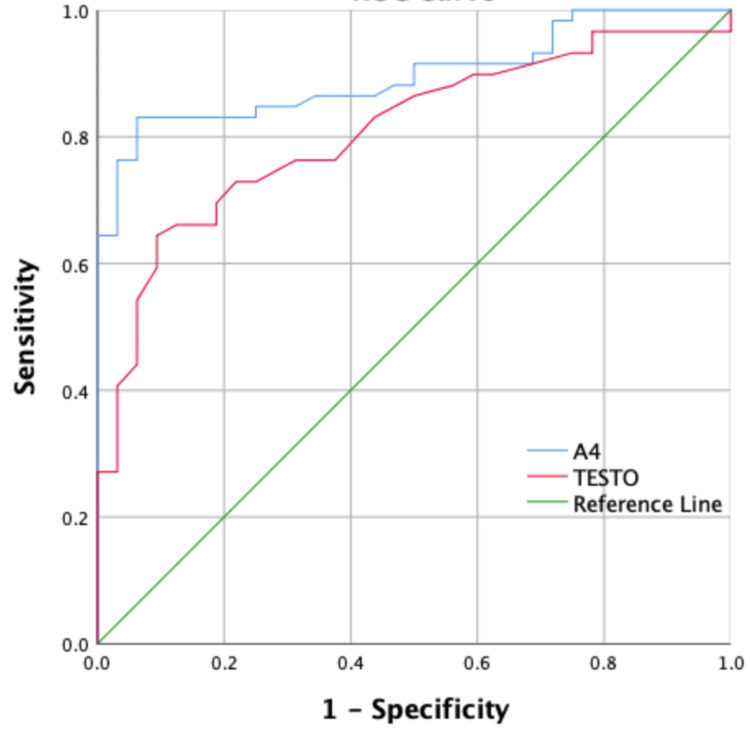
Receiver operating characteristic (ROC) curve for different androgens to diagnose polycystic ovary syndrome (PCOS)

In ROC analysis, A4 had an area under the curve (AUC) of 0.89 (95% CI 0.84 - 0.96) and T had an AUC of 0.81 (95% CI 0.72 - 0.9) (Figure 2). A4 value of 1505 ng/L had 82% sensitivity and 91% specificity while the value of >2000 ng/L had 64% sensitivity and 100% specificity. T at the value of 30 ng/dL had 76% sensitivity and 70% specificity while the value of > 45 ng/dL had 50% sensitivity and 94% specificity. 

## Discussion

In this prospective age- and BMI-matched case-control study in women with PCOS in whom androgens were measured through LC-MS/MS, we gained several insights. Firstly, this was the first study to evaluate the panel of androgens through LC-MS/MS in women with PCOS from India and the ethnic differences in pattern of hyperandrogenemia was realized. Secondly, 40% of women with PCOS phenotypes A-C, who have hyperandrogenemia by definition, had normal T levels. Thirdly, A4 had a greater discriminatory role in diagnosing women with PCOS in general in comparison with T. Lastly, in a subgroup of PCOS without clinical/biochemical hyperandrogenemia (PCOS D), A4 was significantly elevated as compared to controls.

In the past decade, several studies have reported the pattern of hyperandrogenemia of women with PCOS as measured by LC-MS/MS done in different ethnicities [4,8,9]. While the majority of studies investigating the role of measuring androgens with LC-MS/MS in PCOS have shown only marginally better or similar diagnostic ability in comparison with androgens measured through immunoassay, with more refinement and standardization in LC-MS/MS technology, the cost-effectiveness of both modalities has to be clearly studied. There are few advantages of profiling steroids. Firstly, milder forms of CAH may be detected. In a cohort of patients seeking infertility treatment, CAH variants were common in women with PCOS (8.4% versus 3.5% in non-PCOS women) [10]. Secondly, with increasing recognition of 11-keto steroids in reproductive physiology, it may also be incorporated into the profile so that new insights regarding the pathophysiology of PCOS may be discerned. This group of steroids and its research in role of diseases is in the naïve stage but may very well be in common use in the near future [11]. 

PCOS A-C are defined by the presence of hyperandrogenism. The role of total T in detecting hyperandrogenism is well-known to be limited. SHBG is low in hyperandrogenic states and calculating free androgen index requires measuring SHBG which acts as a proxy for free T. Free T was comparable to A4 in detecting hyperandrogenism in women with PCOS [3]. The improvement in diagnosis of hyperandrogenism in PCOS also increased from 80 to 90% in the same study. Rather than measuring T by LC-MS/MS and SHBG as an additional investigation to calculate free T, directly performing the androgen profile by LC-MS/MS would be cost-effective. Moreover, as highlighted in our study, there were few cases involving isolated increases in DHEA with normal T and A4 which would also be captured by androgen profiling. DHEA in isolation had poor diagnostic capability for diagnosing PCOS.

When classified according to Rotterdam criteria, a category of PCOS that does not manifest with hyperandrogenism arises (PCOS-D). Since, by definition, this category has normal androgen levels, research into the role of androgens in this category is lacking. In our study, we found a significant difference in A4 levels, albeit within the normal range, between controls and the PCOS-D category. This is of interest because A4 has been correlated with the metabolic phenotype of PCOS women and severity of clinical phenotype [12,13]. PCOS-D is a relatively less severe phenotype and implications of A4 in this group may be worth investigating.

There were several limitations in the study. Firstly, sample size was not met in the study but the interim analysis showed with the mean difference achieved in both groups for A4 and T, the power of study was >90%. Secondly, SHBG was not measured and subsequently free T was not calculated. Thirdly, the women were representative of only one city and diverse Indian ethnic subgroups were not included. Fourthly, proper cost-benefit analysis could not be performed as data regarding CAH prevalence in Indians were not available.

## Conclusions

In our study, A4 as measured through LC-MS/MS showed higher capability of diagnosing PCOS. A4 in combination with DHEA were elevated in 40% of hyperandrogenic women in the face of normal T. Hence, measuring androgens as a panel has several distinct advantages but the cost-benefit analysis needs to be done for wider adoption of this practice.
